# Correlates of Gross Motor Competence in Children and Adolescents: A Systematic Review and Meta-Analysis

**DOI:** 10.1007/s40279-016-0495-z

**Published:** 2016-02-19

**Authors:** Lisa M. Barnett, Samuel K. Lai, Sanne L. C. Veldman, Louise L. Hardy, Dylan P. Cliff, Philip J. Morgan, Avigdor Zask, David R. Lubans, Sarah P. Shultz, Nicola D. Ridgers, Elaine Rush, Helen L. Brown, Anthony D. Okely

**Affiliations:** 1 Faculty of Health, School of Health and Social Development, Deakin University, Building BC, 221 Burwood Hwy, Burwood, Melbourne, 3125 Australia; 2 Faculty of Social Sciences, Early Start Research Institute, University of Wollongong, Wollongong, Australia; 3 Prevention Research Collaboration, University of Sydney, Sydney, Australia; 4 Faculty of Education and Arts, Priority Research Centre in Physical Activity and Nutrition, University of Newcastle, Newcastle, Australia; 5 Northern NSW Health Promotion, Lismore, Australia; 6 University Centre for Rural Health North Coast, School of Public Health, The University of Sydney, Sydney, Australia; 7 School of Health and Human Sciences, Southern Cross University, Lismore, Australia; 8 School of Sport and Exercise, Massey University, Wellington, New Zealand; 9 Faculty of Health, Centre for Physical Activity and Nutrition Research, Deakin University, Melbourne, Australia; 10 Centre for Child Health Research, Auckland University of Technology, Auckland, New Zealand

## Abstract

**Background:**

Gross motor competence confers health benefits, but levels in children and adolescents are low. While interventions can improve gross motor competence, it remains unclear which correlates should be targeted to ensure interventions are most effective, and for whom targeted and tailored interventions should be developed.

**Objective:**

The aim of this systematic review was to identify the potential correlates of gross motor competence in typically developing children and adolescents (aged 3–18 years) using an ecological approach.

**Methods:**

Motor competence was defined as gross motor skill competency, encompassing fundamental movement skills and motor coordination, but excluding motor fitness. Studies needed to assess a summary score of at least one aspect of motor competence (i.e., object control, locomotor, stability, or motor coordination). A structured electronic literature search was conducted in accordance with the Preferred Reporting Items for Systematic Reviews and Meta-Analyses statement. Six electronic databases (CINAHL Complete, ERIC, MEDLINE Complete, PsycINFO^®^, Scopus and SPORTDiscus with Full Text) were searched from 1994 to 5 August 2014. Meta-analyses were conducted to determine the relationship between potential correlates and motor competency if at least three individual studies investigated the same correlate and also reported standardized regression coefficients.

**Results:**

A total of 59 studies were identified from 22 different countries, published between 1995 and 2014. Studies reflected the full range of age groups. The most examined correlates were biological and demographic factors. Age (increasing) was a correlate of children’s motor competence. Weight status (healthy), sex (male) and socioeconomic background (higher) were consistent correlates for certain aspects of motor competence only. Physical activity and sport participation constituted the majority of investigations in the behavioral attributes and skills category. Whilst we found physical activity to be a positive correlate of skill composite and motor coordination, we also found indeterminate evidence for physical activity being a correlate of object control or locomotor skill competence. Few studies investigated cognitive, emotional and psychological factors, cultural and social factors or physical environment factors as correlates of motor competence.

**Conclusion:**

This systematic review is the first that has investigated correlates of gross motor competence in children and adolescents. A strength is that we categorized correlates according to the specific ways motor competence has been defined and operationalized (object control, motor coordination, etc.), which enables us to have an understanding of what correlates assist what types of motor competence. Indeed our findings do suggest that evidence for some correlates differs according to how motor competence is operationalized.

## Key Points

**Table:** 

Increasing age was the most consistent correlate of all aspects of motor competence.
Aside from age, correlates of motor competence differ according to how motor competence is operationalized.
Investigating correlates of motor skills in children and adolescents is an emerging area with much scope for future investigation.

## Introduction

Motor competence can be defined as a person’s ability to execute different motor acts, including coordination of fine and gross motor skills that are necessary to manage everyday tasks [1]. Gross motor competence in particular plays an important role in growth, development and opportunities to lead an active lifestyle [2]. Gross motor competence is often specified as proficiency in a range of fundamental movement skills (e.g., throwing, catching, running) that are ideally learnt during the preschool and early school years [3–5]. These provide a foundation for children to develop more specialized movement sequences, such as sport-specific [6] (e.g., pitching in baseball) and lifelong physical activity (PA) movement skills (e.g., cycling and swimming) [7]. Fundamental movement skills are often described more precisely as basic stability (e.g., static balance), object control (also termed manipulative, e.g., throwing) or locomotor movements involving two or more body segments, (e.g., jumping) [5]. In this review, the global term “gross motor competence” will be used to reflect the various terminology used in the literature (e.g., fundamental movement/motor skills, stability skills, motor coordination) to define goal-directed human movement [8].

Emerging evidence supports associations between gross motor competence and a range of health outcomes. Children with low levels of gross motor competence tend to be less physically active and have lower levels of cardio-respiratory fitness [9]. A systematic review of 21 studies in children found strong evidence for positive associations between gross motor competency and time spent in PA, and cardio-respiratory fitness, and an inverse association with weight status [2]. More recent reviews have confirmed a positive association between gross motor competence and organized PA [10], and fitness [11]. Furthermore, there is longitudinal evidence that motor competence is important across the developmental lifespan [12]. For instance, higher gross motor competence attenuates the decline in PA levels throughout childhood [13], and motor competency in childhood is associated with higher levels of PA and fitness in adolescence [14, 15]. In addition, longitudinal studies in children have demonstrated that lower motor competence is associated with increased body mass index (BMI) over time [16, 17].

Despite the health benefits associated with gross motor competence, motor competence in children and adolescents is low [9, 18, 19], with only 50 % of children demonstrating competency in a broad range of skills [20–23]. While recent papers [24] and systematic reviews [25–28] indicate interventions can improve gross motor competence in both children and adolescents, published manuscripts lack important details (such as intervention intensity, duration, fidelity and characteristics of facilitators and participants) [27]. It remains unclear from these studies which correlates should be targeted to ensure interventions are optimized, and whether or not, and for whom, targeted and tailored interventions should be developed.

Understanding these important aspects of intervention development requires systematically reviewing the correlates of gross motor competence in children and adolescents. This will help to identify potential mechanisms of change by identifying the factors that are likely to make a difference and also target specific groups for intervention [29]. Ecological models are useful in framing potential influencing factors of health behavior (e.g., PA) [30] as they emphasize the environmental contexts of the behavior as well as the social and psychological influences. This can lead to an in-depth understanding of the multiple spheres of influence on behavior and can help guide intervention development.

Previous reviews of the pediatric correlates of motor competence have been limited by focusing only on fundamental movement skills [2] rather than using a broader definition of gross motor competence, examining only certain age groups (e.g., preschool) [31] and only documenting positive associations [31] (and not including null or negative associations). The current review will expand upon previous reviews [2, 31] in several important ways. Given that gross motor competence is important across the developmental lifespan [12], the age range from early childhood (age 3 years) to adolescence (up to age 18 years) will be reviewed. Furthermore, the review will identify gross motor competence as the outcome of interest and will endeavor to find which factors are reported as potential correlates of motor competence, using an ecological framework. Correlates will be categorized under five broader categories, namely (i) biological and demographic factors; (ii) behavioral attributes and skills; (iii) cognitive, emotional and psychological factors; (iv) cultural and social factors; and (v) physical environmental factors, as per a previous review on key correlates of PA [32], to understand the potential correlates of motor competency.

Whilst it is acknowledged that the association between gross motor competence and factors such as PA are likely to be reciprocal [12, 33], focusing on motor competence as the outcome (or dependent) variable will enable an examination of those factors that are potentially modifiable correlates of motor competence in young people. This will make the findings important and relevant to interventionists seeking to find ways of improving motor competence of children. This will also ensure this review does not replicate previous reviews that have examined the association between motor competence and PA or fitness [2, 10, 11]. Further, this review will also document null and negative correlates of gross motor competence, which will further help to isolate factors that are not important to target.

## Methods

### Identification of Studies

A structured electronic literature search was conducted in accordance with the Preferred Reporting Items for Systematic Reviews and Meta-Analyses (PRISMA) statement [34]. Six electronic databases (CINAHL Complete, ERIC, MEDLINE Complete, PsycINFO^®^, Scopus and SPORTDiscus with Full Text) were searched from 1994 to 5 August 2014. Five of these databases (CINAHL Complete, ERIC, MEDLINE Complete, PsycINFO^®^ and SPORTDiscus with Full Text) were accessed through the EBSCOhost platform.

The following search strings were used: (“motor skill*” OR “movement skill*” OR “motor development” OR “gross motor” OR “motor performance” OR “motor abilit*” OR “object manipulation” OR “motor coordination” OR “actual competence” OR “object control” OR “locomotor skill*” OR “motor proficiency” OR “motor competence”) AND (preschool* OR kindergarten* OR child* OR adolescen* OR student* OR teen* OR youth) AND (correlate* OR determinant* OR predictor* OR relationship* OR association* OR difference*). An additional line of search terms was added to exclude studies with a focus on children and/or adolescents with a physical or cognitive impairment. These were as follows: AND NOT disabilit* OR disorder* OR impair* OR “cerebral palsy” OR autis*.

These strings were further limited to participants aged 3–18 years and English language. Only articles published in peer-reviewed journals were considered. Reviews, conference proceedings, and abstracts were not included. In addition to identifying studies through the database search, studies from authors’ own bibliographic libraries were assessed for possible inclusion. After duplicates were removed, studies were initially assessed by screening titles and abstracts. If suitability could not be determined during this process, full-text articles were accessed and compared against inclusion criteria. The reference lists of retrieved full-text articles and other systematic reviews were also examined for relevant studies.

### Selection Criteria

Two authors (SKL, SLCV) independently assessed the eligibility of studies for inclusion using the criteria below. Two other authors (LMB and ADO) were consulted when agreement could not be reached.

1. Participants were aged 3–18 years. The infant and toddler period were excluded so as to enable focus on motor competence rather than motor milestones or early developmental aspects. Studies with a focus on children and/or adolescents with a physical or cognitive impairment were excluded (e.g., cerebral palsy). Studies targeting overweight/obese children or children from schools in disadvantaged areas were included, but not those where study inclusion criteria specified that participants had developmental coordination delays.
2. Studies assessed gross motor competence. Motor competence was specified broadly as gross motor skill competency, encompassing fundamental movement skills and motor coordination.
3. Studies that used measurement batteries that were defined as “motor fitness” were excluded. Whilst physical fitness components such as cardiorespiratory endurance, body composition, muscular strength, endurance, and flexibility are sometimes termed “motor fitness” or “motor ability” [35], they were not considered as motor competence assessments for this review. Similarly, other performance-related components of fitness, such as agility [which can be defined as “a rapid whole-body movement with change of velocity or direction in response to a stimulus” [36] (p. 922)], were not considered as assessments of motor competence unless there was a clear distinction between the components being analyzed and discussed as aspects of fitness and those being analyzed and discussed as aspects of motor competence.
4. Studies that used measurement batteries that included fine motor skills as part of a composite score were excluded to preserve internal validity {e.g., the Motoriktest für vier- bis sechsjährige Kinder 4–6 [37] and McCarron Assessment of Neuromuscular Development (MAND) [38]}, unless analysis was conducted without the inclusion of fine motor skills. For some instruments, however, that assessed fine motor skills, one or more subtests may have met our inclusion criteria. For the Bruininks-Oseretsky Test of Motor Proficiency (BOTMP) [39], we only included the balance assessment, and only if more than two tests were used for this subtest. Other subtests in this assessment were excluded. Bilateral coordination (even in the short form of the assessment) contains both gross and fine motor elements. Manual coordination is a combination of two subtests: manual dexterity (which assesses fine motor skills) and upper limb coordination. The body coordination subtest includes bilateral coordination (which assesses fine motor skills). The agility component was excluded as this did not meet our criteria for a gross motor skill. For the Movement Assessment Battery for Children (M-ABC) [1], we included the balance subtest and the ball skills subtest. For the Peabody Developmental Motor Scales, Second Edition (PDMS–2) [40], we included the gross motor score and the subtests for object control, locomotor and stability.
5. Studies needed to assess a summary score of at least one aspect of gross motor competence. This could be an object control, locomotor, stability, or motor coordination summary score. At least two skill assessments needed to be included to make up a summary score. Studies that analyzed individual skills separately as the outcome variable were not included because the purpose of the review was to assess the factors that contributed to motor competence (or aspects of motor competence, defined above) more generally, rather than the factors that contributed to competency in one particular skill.
6. Studies presented a quantitative analysis of the association between a potential correlate and at least one aspect of gross motor competence as the outcome. Studies where gross motor competence was not treated as the outcome variable in analysis were excluded (i.e., correlation analysis). Studies that may have reported associations but still treated gross motor competence as the outcome or criterion variable were included.
7. Studies identified a potential correlate of gross motor competence that was not related to improvement as part of an intervention. For instance baseline associations would be potentially included, but not associations due to the impact of an intervention.

### Criteria for Risk of Bias Assessment

Four authors (DPC, LLH, AZ, PJM) independently assessed the risk of bias in the studies that met the inclusion criteria. The criteria for assessing the risk of bias in the studies were adapted from the Strengthening the Reporting of Observation Studies in epidemiology (STROBE) statement [41] and previous reviews in similar areas [2, 27]. Four criteria were identified as being of most importance to this current review. These criteria were:

Were the participants likely to be representative of the population (i.e., country, state/region level)? Were schools or students randomly selected or were other data provided to indicate population representativeness?
Of those who consented to the study, did an adequate proportion have complete data for the outcome and *all* correlates of interest (i.e., no more than 20 % of data was missing from a cross-sectional study and no more than 30 % for a longitudinal study)?
(a) Did the study report the sources and details of motor competence assessment and were valid measures of motor competence used (validation in same age group published or validation data provided in the manuscript)?
(b) Did the study report adequate reliability of motor competence assessment [i.e., intra-class correlation (ICC) (or similar) ≥0.60] [42]? For studies that used process-oriented motor competence instruments (i.e., were concerned with the process or execution of the skill movement), adequate inter-rater reliability needed to be reported [i.e., ICC (or similar) ≥0.60] in addition to the above validity and reliability measures.
Did the study report the sources and details of assessment of potential correlates and did all of the correlates of interest have acceptable validity and/or reliability? Acceptable validity was defined as >0.40, as per Brown et al. [42].

Initially five articles were sent to all authors to assess. After this assessment, any differences in risk of bias assessment were resolved via teleconference. Criteria were further refined, and two more studies were sent to all four authors. After a further discussion, the criteria were finalized and the remaining studies (including the five previously assessed) were divided among the four authors to score. Another author (SPS) then checked all studies to ensure consistency across the four raters in terms of criterion 3 only. This criterion was selected for an additional check because many studies used similar motor competence assessment instruments and we wanted to ensure raters had been comparable. Any differences at this point were resolved within the group of five authors facilitated by author LMB. Each criterion was scored as “yes” (a tick), “no” (a cross), “unclear” (?).

### Categorization of Variables and Level of Evidence

Each correlate was summarized according to the state of evidence for that correlate. As per the review by Sallis and colleagues in 2000 [43], the percentages in parentheses refer to the number of associations supporting the expected association divided by the total number of associations for the variable. Based on the percentage of findings supporting the association, the variable was classified as no association (0–33 %), written as “0”; indeterminate/inconsistent (34–59 %), written as “?”; or a positive “+” or negative “−” association (≥60 %). When four or more studies found an association, it was classified as “++” or “− −” accordingly. When findings differed by sex, or age/year, these were noted as “male” or “female” or the age/year group. Summary codes were then based on the analysis that either supported the expected direction (either positive or negative) or did not (non-correlate). This meant that if a study found a positive association in one age group between a correlate and an aspect of motor competence and no association in another age group, the findings were counted in the summary codes as one study for and one study against. Some variables that were conceptually similar were combined for the purpose of summary codes if there were not enough studies to examine the variables individually (e.g., age and school year group).

### Meta-Analyses

Meta-analyses were conducted to determine the relationship between gross motor competency and potential correlates using comprehensive meta-analysis software, version 2 for Windows (Biostat company, Englewood, NJ, USA) [44] with random effects models. Meta-analyses were conducted if at least three individual studies reported standardized coefficients. When studies compared reported multiple coefficients from the same study sample collected over different time points, the sample size was divided to avoid double counting. Heterogeneity was determined by Cochrane’s *Q* statistic and *I* ^2^ values (values of 25, 50 and 75 were considered to indicate low, moderate and high heterogeneity, respectively) [45]. Publication bias was analyzed using Rosenthal’s classic fail safe *N* [46]. Correlations were interpreted as follows: 0–0.19 (no correlation), 0.2–0.39 (low correlation), 0.4–0.59 (moderate correlation), 0.6–0.79 (moderately high correlation) and ≥0.8 (high correlation) [47].

## Results

### Overview of Studies

**Fig. 1:**
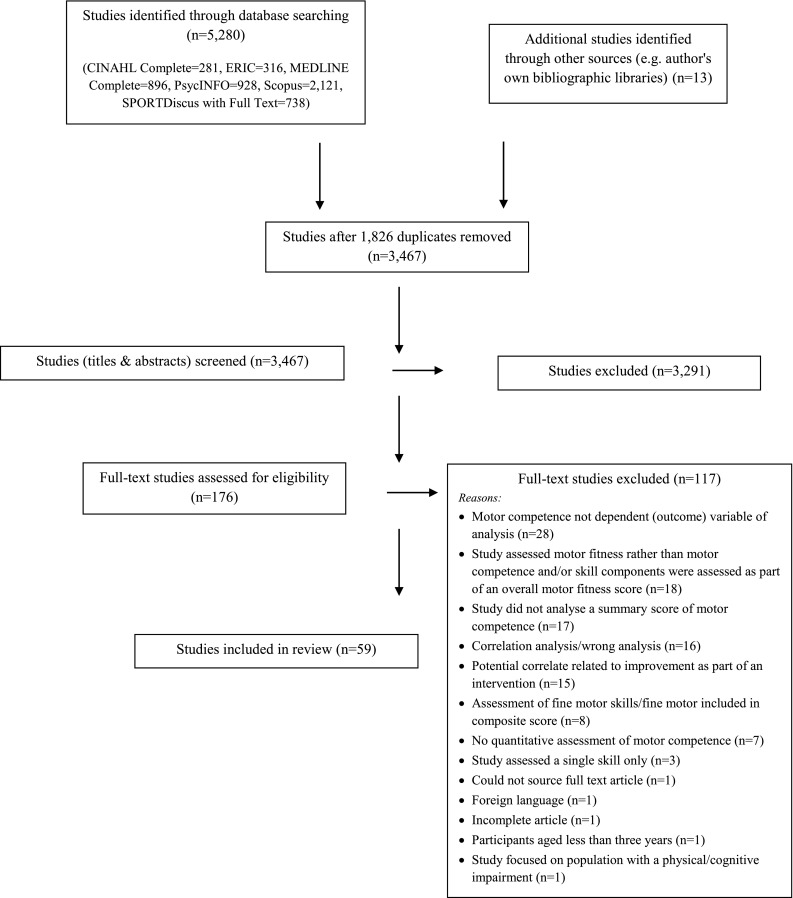
Study progression during inclusion/exclusion

A total of 59 studies were identified (Fig. 1), published between 1995 and 2014. One author extracted descriptive data from the studies (HLB), and this was checked by two other authors (SKL and SLCV). Studies reflected the full range of ages, with one study in the birth to preschool age group [48] (included due to the upper age range), 19 [22, 49–66, 93] in the preschool age group (defined as prior to school, 3–5 years), 29 [18, 67–92, 94, 95] in children (defined as primary, elementary school age), four in adolescents [33, 96–98], and five that covered a range of ages: preschool and children [23, 99]; children and adolescents [19, 100, 101].

Most studies were conducted in the USA [18, 22, 51, 53, 58, 61–63, 66, 82, 87] and Australia [33, 49, 50, 70, 75, 76, 95, 96, 98, 100]. A total of 21 studies were conducted across Europe (Belgium [71–73, 90, 91], Finland [48, 88, 97, 99], Portugal [19, 64, 80], Denmark [59, 84], Greece [89, 93], Germany [101], Ireland [81], Italy [57], Northern Ireland [83] and Norway [92]), eight in Asia (Hong Kong [52, 55, 69], Bangladesh [85], India [67], Indonesia [68], Japan [54] and Pakistan [74]), five in Canada [56, 65, 77, 79, 94], two in Brazil [23, 60] and a single study each from Israel [86] and South Africa [78]. Most study designs were cross-sectional [18, 19, 22, 23, 33, 49, 50, 52–71, 74–80, 83–90, 92–95, 98, 100, 101]. There were eight longitudinal studies [48, 72, 73, 81, 82, 91, 96, 97] and two randomized controlled trials (RCTs) [51, 99]. One RCT was included because they analyzed baseline data [51] and the other because they analyzed midline data only [99]. The sample sizes ranged from 34 [63] to >5000 [19]. More than half the studies had samples of <300 (Tables 1, 2).

**Table 1:** Descriptive information of included studies (ordered alphabetically)

Study	Year	Country	Sample size	Sex (B, G)	Age (mean ± SD)	Age group	Design
Abbas et al. [67]	2011	India	197	B 99, G 98	9½–14½ y	Children	Cross-sectional
Bakhtiar [68]	2014	Indonesia	67	B 28, G 39	6.55 ± 0.25 y	Children	Cross-sectional
Barnett et al. [96]	2010	Australia	266	B 48.1 %, G 51.9 %	T1: 10.06 ± 0.63 y T2: 16.44 ± 0.6 4y	Adolescents	Longitudinal
Barnett et al. [33]	2011	Australia	215	B 48.4 %, G 51.6 %	16.4 ± 0.6 y	Adolescents	Cross-sectional
Barnett et al. [50]	2012	Australia	53	B 41 %, G 59 %	4.15 ± 0.72 y	Preschool	Cross-sectional
Barnett et al. [49]	2013	Australia	76	B 34, G 42	4.1 ± 0.68 y	Preschool	Cross-sectional
Bellows et al. [51]	2013	USA	263	B NR, G NR	C: 51.5 ± 6.6 mo I: 53 ± 6.8 mo	Preschool	Randomized controlled trial (baseline data used)
Choi Tse [69]	2004	Hong Kong	90	B 45, G 45	6–8 y	Children	Cross-sectional
Chow and Chan [52]	2011	Hong Kong	239	B 121, G 118	3.6 ± 0.2 y	Preschool	Cross-sectional
Cohen et al. [70]	2014	Australia	460	B 46 %, G 54 %	8.5 ± 0.6 y	Children	Cross-sectional
D’Hondt et al. [71]	2009	Belgium	117	B 57, G 60	5–10 y	Children	Cross-sectional
D’Hondt et al. [72]	2013	Belgium	100	B 52 %, G 48 %	T1: 8.2 ± 1.2 y T2: NR	Children	Longitudinal
D’Hondt et al. [73]	2014	Belgium	T1: 2517 T2: 754	T1: B 52.8 %, G 47.2 % T2: B 50.8 %, G 49.2 %	T1: 5–13 y T2: 7–13 y	Children	Longitudinal
Erwin and Castelli [18]	2008	USA	180	B 87, G 93	10.45 ± 0.7 8y	Children	Cross-sectional
Goodway and Rudisill [53]	1997	USA	59	B 30, G 29	4.74 ± 0.31 y	Preschool	Cross-sectional
Goodway et al. [22]	2010	USA	469 (MW = 275, SW = 194)	MW: B 143, G 132 SW: B 95, G 99	MW: 54.80 mo SW: 56.37 mo	Preschool	Cross-sectional
Habib et al. [74]	1999	Pakistan	180	B 90, G 90	5–13 y	Children	Cross-sectional
Hume et al. [75]	2008	Australia	248	B 123, G 125	B: 10.1 ± 0.44 y G: 10.0 ± 0.28 y	Children	Cross-sectional
Iteya et al. [54]	1995	Japan	273	Foot laterality: B 93, G 42 Hand laterality: B 78, G 60	Foot laterality: 5.3 y Hand laterality: 5.5 y	Preschool	Cross-sectional
Jaakkola and Washington [97]	2013	Finland	152	B 86, G 66	T1: 13 y T2: NR T3: NR	Adolescents	Longitudinal
Jones et al. [76]	2010	Australia	1299	B 52 %, G 48 %	6.35 ± 1.07 y	Children	Cross-sectional
Junaid and Fellowes [77]	2006	Canada	103	B 60, G 43	7–8 y	Children	Cross-sectional
Kemp and Pienaar [78]	2013	South Africa	816	B 419, G 397	6.84 ± 0.39 y	Children	Cross-sectional
Lam and Schiller [55]	2001	Hong Kong	320	B 149, G 171	5–6 y	Preschool	Cross-sectional
Larouche et al. [79]	2014	Canada	491	B 43.6 %, G 56.4 %	Grade 4–6	Children	Cross-sectional
Laukkanen et al. [99]	2014	Finland	84	B 38, G 46	Preschool B: 5.92 ± 0.45 y Preschool G: 5.95 ± 0.47 y Primary B: 7.93 ± 0.34 y Primary G: 8.06 ± 0.15 y	Preschool and children	Randomized controlled trial (midline data used)
LeGear et al. [56]	2012	Canada	260	B 52 %, G 48 %	5 y 9 mo	Preschool	Cross-sectional
Lopes et al. [80]	2012	Portugal	213	B 103, G 110	9.46 ± 0.43 y	Children	Cross-sectional
Lopes et al. [19]	2012	Portugal	7175	B 3616, G 3559	6–14 y	Children and adolescents	Cross-sectional
MacCobb et al. [81]	2005	Ireland	76	B 38, G 38	8–10 y	Children	Longitudinal
McKenzie et al. [82]	2002	USA	207	B 104, G 103	T1: 4 y T2: 5 y T3: 6 y	Children	Longitudinal
McPhillips and Jordan-Black [83]	2007	Northern Ireland	515	B 283, G 232	Year 1: 57.4 ± 3.6 mo Year 4: 101.4 ± 4.5 mo	Children	Cross-sectional
Morano et al. [57]	2011	Italy	80	B 38, G 42	4.5 ± 0.5 y	Preschool	Cross-sectional
Morrison et al. [84]	2012	Denmark	498	B 265, G 233	B: 6.8 ± 0.4 y G: 6.7 ± 0.4 y	Children	Cross-sectional
Nervik et al. [58]	2011	USA	50	B 26, G 24	53 ± 10.5 mo	Preschool	Cross-sectional
Okely et al. [100]	2004	Australia	4268	B 2295, G 1973	Grade 4, 6, 8, 10	Children and adolescents	Cross-sectional
Okely et al. [98]	2001	Australia	2026	B 1081, G 945	Year 8: 13.3 y Year 10: 15.3 y	Adolescents	Cross-sectional
Olesen et al. [59]	2014	Denmark	607	B 299, G 308	5.8 ± 0.3 y	Preschool	Cross-sectional
Parvez et al. [85]	2011	Bangladesh	303	B 50 %, G 50 %	9.6 ± 0.7 y	Children	Cross-sectional
Queiroz et al. [60]	2014	Brazil	LOC SP: 54 LOC NSP: 54 OC SP: 37 OC NSP: 37	LOC SP: B 30, G 24 LOC NSP: B 30, G 24 OC SP: B 17, G 20 OC NSP: B 17, G 20	LOC SP: 60.0 ± 8.7 mo LOC NSP: 59.4 ± 8.1 mo OC SP: 60.9 ± 7.9 mo OC NSP: 60.7 ± 7.9 mo	Preschool	Cross-sectional
Ratzon et al. [86]	2000	Israel	114 (children born to diabetic mothers = 57, children in control group = 57)	Children born to diabetic mothers: B 51 %, G 49 % Children in control group: B 56 %, G 44 %	Children born to diabetic mothers: 8.09 ± 1.77 y Children in control group: 8.29 ± 1.78 y	Children	Cross-sectional
Roberts et al. [61]	2012	USA	4650	B 2150, G 2500	5 y 3 mo ± 4 mo	Preschool	Cross-sectional
Robinson [62]	2010	USA	119	B 65, G 54	4 ± 0.55y	Preschool	Cross-sectional
Robinson et al. [63]	2012	USA	34	B 12, G 22	57 ± 6.31 mo	Preschool	Cross-sectional
Roeber et al. [87]	2012	USA	67 (adopted = 33, not adopted = 34)	Adopted: B 16, G 17 Not adopted: B 21, G 13	Adopted: 10 y 9 mo ± 2 y 2 mo Not adopted: 11 y 2 mo ± 2 y 1 mo	Children	Cross-sectional
Saraiva et al. [64]	2013	Portugal	367	B 172, G 195	53 ± 9.6 mo	Preschool	Cross-sectional
Slotte et al. [88]	2014	Finland	304	B 153, G 151	8.6 ± 0.2 y	Children	Cross-sectional
Spessato et al. [23]	2013	Brazil	1248	B 641, G 607	3–10 y	Preschool and children	Cross-sectional
Temple et al. [65]	2014	Canada	74	B 41, G 33	5 y 11 mo ± 4 mo	Preschool	Cross-sectional
Tsapsakidou et al. [89]	2014	Greece	100	B 54, G 46	8–9 y	Children	Cross-sectional
Vandendriessche et al. [90]	2012	Belgium	1955	B 52 %, G 48 %	6–11 y	Children	Cross-sectional
Vandorpe et al. [91]	2012	Belgium	371	NR	T1: 8.3 ± 1.1 y T2: 10.3 ± 1.1 y	Children	Longitudinal
Vedul-Kjelsås et al. [92]	2013	Norway	67	B 39, G 28	B: 11.50 ± 0.26 y G: 11.40 ± 0.26 y	Children	Cross-sectional
Venetsanou and Kambas [93]	2011	Greece	283	B 145, G 138	61.77 ± 5.43 mo	Preschool	Cross-sectional
Viholanen et al. [48]	2006	Finland	130	B 70, G 60	3.5 y	Birth to preschool	Longitudinal
Woll et al. [101]	2013	Germany	4519	B 2,310, G 2,209	4–17 y	Children and adolescents	Cross-sectional
Woodard and Yun [66]	2001	USA	138	B 65, G 73	5.3 y	Preschool	Cross-sectional
Wright and Bos [94]	2012	Canada	84	B 44, G 40	8–11 y	Children	Cross-sectional
Ziviani et al. [95]	2009	Australia	124	B 55, G 69	6–12 y	Children	Cross-sectional

*B* boy, *C* control, *G* girl, *I* intervention, *LOC* locomotor skills, *mo* months, *MW* Midwestern, *NR* not reported, *NSP* no sports practice, *OC* object control skills, *SP* sports practice, *SW* Southwestern, *T1* time point 1, *T2* time point 2, *T3* time point 3, *y* years

**Table 2:** Studies categorized by sample size

Total sample	No. of studies	References
<100	14	[49, 50, 53, 57, 58, 63, 65, 68, 69, 81, 87, 92, 94, 99]
100–199	14	[18, 48, 60, 62, 66, 67, 71, 72, 74, 77, 86, 89, 95, 97]
200–299	10	[33, 51, 52, 54, 56, 75, 80, 82, 93, 96]
300–399	5	[55, 64, 85, 88, 91]
400–499	4	[22, 70, 79, 84]
500–999	3	[59, 78, 83]
1000–2999	4	[23, 76, 90, 98]
3000–5000	4	[61, 73, 100, 101]
>5000	1	[19]

A range of instruments were used to assess gross motor competency. More than half (33 studies) used product-oriented assessments (these are concerned with the outcome of movement, such as number of repetitions or whether the ball hits a target). The Körperkoordinationtest für Kinder (KTK) was used in eight studies [19, 72, 73, 80, 84, 90, 91, 101]. Several product-oriented assessments that use composite gross and fine motor batteries were included, with gross motor competence analyzed separately (as per our inclusion criteria): the BOTMP first edition, seven studies [55, 67, 74, 81, 86, 87, 93] and second edition, one study [85]; the M-ABC (six studies [48, 71, 77, 83, 92, 95]); and the PDMS–2 (three studies [51, 58, 64]). Five other studies used product-oriented tests particular to one study only [18, 54, 82, 94, 97], and three studies used a combination of different product assessments [59, 61, 99].

A total of 24 studies used process-oriented assessments. The most commonly used was the Test of Gross Motor Development (TGMD): first edition, three studies [53, 57, 66] and second edition, 15 studies [22, 23, 49, 50, 52, 56, 60, 62, 63, 65, 68–70, 88, 89]. Australian resources were used in six studies (Department of Education Victoria [75, 98, 100] and Get Skilled Get Active [33, 76, 96]; three studies each). Two studies used both process and product assessment elements [78, 79].

Correlates were classified into the following five categories: (1) biological and demographic factors; (2) behavioral attributes and skills; (3) cognitive, emotional and psychological factors; (4) cultural and social factors; and (5) physical environmental factors. In total, 49 correlates were assessed, with most studies assessing one (21 studies), two (21 studies) or three correlates (eight studies), and nine studies assessing between four and 12 correlates.

### Overview of Study Risk of Bias

Study risk of bias is presented in Table 3 and shows that nearly one-third of studies (32 %) had samples that could be classed as representative of the study population, 58 % of studies had minimal missing data, 86 % used valid measures of gross motor competence, and 73 % used reliable measures of motor competence. Most studies assessed potential correlates in a valid and reliable manner.

**Table 3:** Risk of bias results

Study details		Study quality				Correlates assessed and quality				
Study	Year	Representative sampling	Minimal missing data	Valid FMS	FMS reliabilities ≥ 0.60	Number of correlates	Age	Sex	BMI	Other correlates
Abbas et al. [67]	2011	✓	?	✓	✓	2	✓	✓		
Bakhtiar [68]	2014	✓	?	✓	?	1		✓		
Barnett et al. [96]	2010	✘	✘	✘	✓	2	✓	✓		
Barnett et al. [33]	2011	✓	✓	✘	✓	2				MVPA (✓), perceived sports competence (✓)
Barnett et al. [50]	2012	✘	✘	✓	✓	5	✓	✓		PA (✓), non-interactive games (✘), interactive games (✘)
Barnett et al. [49]	2013	✘	✘	✓	✓	12	✓	✓		MVPA (✓), unstructured activities (✘), swimming lessons (✘), dance classes (✘), kindy gym classes (✘), parent child interaction (✘), parent MPA/VPA (✘), parent skill confidence (✘), visits to play spaces (✘), toys home equipment (✘)
Bellows et al. [51]	2013	✓	✓	✓	✓	1				Steps (✓)
Choi Tse [69]	2004	✘	✓	✓	✓	1		✓		
Chow and Chan [52]	2011	✘	✓	✓	✓	2		✓		Preschool size (✓)
Cohen et al. [70]	2014	✓	✓	✓	✓	1		✓		
D’Hondt et al. [71]	2009	✘	✓	✓	✓	2			✓	SES/parental education (✓)
D’Hondt et al. [72]	2013	✓	✓	✓	✓	2			✓	Organized sport (✘)
D’Hondt et al. [73]	2014	✓	✘	✓	✓	3	✓		✓	PA (?)
Erwin and Castelli [18]	2008	✘	✘	✓	✓	6	✓	✓		Ethnicity (✓), school year (✓), fitness (✓), strategic knowledge (?)
Goodway and Rudisill [53]	1997	✘	✓	✓	✓	1		✓		
Goodway et al. [22]	2010	✘	✓	✓	✓	2		✓		Region (✓)
Habib et al. [74]	1999	✘	?	✓	✓	3	✓	✓		SES (✓)
Hume et al. [75]	2008	✘	✘	✓	✓	2		✓	✓	
Iteya et al. [54]	1995	✘	✓	?	?	1				Limb laterality (✘)
Jaakkola and Washington [97]	2013	✘	✓	✓	?	3	✓	✓		PA (?)
Jones et al. [76]	2010	?	✓	✘	?	1			✓	
Junaid and Fellowes [77]	2006	✘	?	✓	✓	1		✓		
Kemp and Pienaar [78]	2013	✓	✓	✓	?	1			✓	
Lam and Schiller [55]	2001	?	✓	?	?	1		✓		
Larouche et al. [79]	2014	✘	✘	✓	✘	1		✓		
Laukkanen et al. [99]	2014	✘	✓	✓	✓	2	✓	✓		
LeGear et al. [56]	2012	✘	✓	✓	✓	1		✓		
Lopes et al. [80]	2012	✘	✘	✓	✓	2		✓		Sedentary behavior (✓)
Lopes et al. [19]	2012	✘	?	✓	✓	2		✓	✓	
MacCobb et al. [81]	2005	✘	✘	✓	✓	6				Birth weight (✓), APGAR at 5 min (✓), Bailey infant behavior (✓), Bailey mental (✓), NBAS motoric cluster (✓), Bailey motor (✓)
McKenzie et al. [82]	2002	✘	✘	✘	✓	2		✓		Ethnicity (✓)
McPhillips and Jordan-Black [83]	2007	✘	✓	✓	✓	2		✓		Disadvantage (✓)
Morano et al. [57]	2011	✘	✓	✓	?	2		✓	✓	
Morrison et al. [84]	2012	✘	✘	✓	?	3		✓		PA (✓), body fat (skinfolds) (?)
Nervik et al. [58]	2011	✘	✘	✘	✓	3	✓	✓	✓	
Okely et al. [100]	2004	✓	✓	✓	✓	2			✓	Waist (✓)
Okely et al. [98]	2001	✓	✓	✓	✓	2	✓	✓		
Olesen et al. [59]	2014	✓	✓	✓	✓	1		✓		
Parvez et al. [85]	2011	✘	✓	✓	✘	3				Arsenic (✓), manganese (✓), selenium (✓)
Queiroz et al. [60]	2014	✓	✓	✓	✓	2		✓		Sport practice (✓)
Ratzon et al. [86]	2000	✘	✓	✓	✓	1				Maternal diabetes (✓)
Roberts et al. [61]	2012	✓	✘	✓	✘	1			✓	
Robinson [62]	2010	✘	✓	✓	✓	1		✓		
Robinson et al. [63]	2012	✘	✓	✓	✓	1		✓		
Roeber et al. [87]	2012	✘	✓	✓	✓	4	✓			Adoption status (✓), time living in USA (✓), Time spent institutionalized before adoption (✓)
Saraiva et al. [64]	2013	✘	?	✓	✓	4	✓	✓	✓	Height (✓)
Slotte et al. [88]	2014	✓	✘	✓	?	5		✓	✓	Waist (✓), height (✓), body fat (✓)
Spessato et al. [23]	2013	✘	?	✓	✓	2	✓	✓		
Temple et al. [65]	2014	✘	✓	✓	?	5	✓	✓		Active physical recreation (?), participation in PA (?), organized sports (?)
Tsapsakidou et al. [89]	2014	✓	✓	✓	✓	5		✓		Maternal education (✓), paternal education (✓), SES (✓), sport participation (✓)
Vandendriessche et al. [90]	2012	✓	✓	✓	✓	3	✓			SES (✓), sport participation (✓)
Vandorpe et al. [91]	2012	✓	✘	✓	✓	1				Organized sport (✓)
Vedul-Kjelsås et al. [92]	2013	✘	✓	✓	✓	1		✓		
Venetsanou and Kambas [93]	2011	✓	✓	✓	✓	2	✓	✓		
Viholanen et al. [48]	2006	✘		✓	✓	2				Early body (✓), hand control (✓)
Woll et al. [101]	2013	✓	?	✓	✘	1		✓		
Woodard and Yun [66]	2001	✘	✓	✓	✘	2		✓		Group/SES (✓)
Wright and Bos [94]	2012	✘	✓	?	✓	3	✓	✓	✓	
Ziviani et al. [95]	2009	✘	✘	✓	?	1		✓		
Total		32.2 % (19/59)	57.6 % (34/59)	86.4 % (51/59)	72.9 % (43/59)		18	42	14	

✓ met criteria, ✘ did not meet criteria, ? unclear whether it met criteria, *APGAR* Appearance, Pulse, Grimace, Activity and Respiration, *BMI* body mass index, *FMS* fundamental movement skills, *MPA* moderate physical activity, *MVPA* moderate to vigorous physical activity, *NBAS* Brazelton Neonatal Behavioral Assessment Scale, *PA* physical activity, *SES* socioeconomic status, *VPA* vigorous physical activity

### Biological and Demographic Correlates

The most commonly investigated biological and demographic correlates were sex (42 studies), age/grade (18 studies) and BMI (14 studies) (Table 4). There was strong evidence that being male was a positive correlate of object control competence and of motor coordination. The association between composite skill scores was indeterminate for boys, and there was no evidence that the sex of a child was associated with locomotor competence. There was inconsistent evidence for “being female” as associated with stability.

There was strong evidence for age (or grade) as a positive correlate of object control, locomotor skills and stability. There was inconsistent evidence for age as a correlate of motor coordination and skill composite.

There was strong evidence that higher BMI was negatively correlated with motor coordination and skill composite, whilst there was moderate evidence for stability. There was no evidence for BMI being negatively associated with object control skills and indeterminate evidence for locomotor skills. Similarly, other measures of adiposity, including higher waist circumference and percentage body fat, were negatively correlated with motor competence.

The socioeconomic background of the child was investigated in seven studies [22, 66, 71, 74, 83, 89, 90] and showed inconsistent findings. A higher socioeconomic background was positively associated with locomotor, stability and skill composite, but one study showed a confounding effect with school year, with socioeconomic background positively influencing younger children but not older children’s object control and stability [83]. Ethnicity was assessed in two studies, with both showing no association with motor composite score [18, 82]. Limb laterality (non-correlate) [54] and fitness (positive association) [18] were only assessed by one study each.

### Behavioral Attributes and Skills Correlates

In terms of behavioral attributes and skills, different categories of PA and sport engagement constituted the majority of investigations in this category. Studies investigating PA were grouped together unless the particular type of PA was specified (e.g., dance, swimming). This resulted in studies addressing organized PA and sport, non-organized PA, and PA according to intensity being summarized together. There was inconsistent evidence for PA being a correlate of object control or locomotor skills. Only one study investigated PA as a correlate of stability competence [51]. There was, however, evidence for PA as a positive correlate of motor coordination [72, 90, 91] and skill composite [51, 84, 97]. Different types of classes (dance, “kindy gym,” swimming) were investigated in one study, so a summary could not be calculated [49].

Sedentary time was only investigated in one study, with less time spent sedentary associated with better motor competence [80]. Early body and hand control were investigated in only one study, which found a positive result for body control and a negative result for hand control [48]. Likewise, the use of interactive and non-interactive electronic games was only investigated by one study, with a positive result for object control competence and no association for locomotor competence [50].

### Evidence for Other Factors as Correlates of Motor Competence

Only one cognitive factor [18] and one psychological factor were investigated [33], with mixed results. One study examined the association between stability and a range of infant measures (study included because of the upper age range), including APGAR (Appearance, Pulse, Grimace, Activity and Respiration) and mental and motor development (Bayley Scales of Infant Development), with equivocal findings [81]. Similarly the studies that assessed a range of cultural and social factors (e.g., adoption status, parent skill confidence) [49, 86, 87, 89] and/or physical environmental factors (e.g., arsenic exposure, playground size at school) [49, 52, 85] produced mixed results. No summary scores could be calculated for these factors because of the lack of studies assessing any one particular variable.

**Table 4:** Correlates summary

Correlate	Association	Object control		Locomotor		Stability		Motor coordination		Skill composite	
		Evidence		Evidence		Evidence		Evidence		Evidence	
**Biological and demographic factors**											
Age			++		++		++		?		?
Age	**+**	[23, 49, 50, 64, 96]	5/7 (71 %)	[23, 49, 50, 64, 96]	5/6 (83 %)	[64, 67, 74, 93]	4/5 (80 %)	[90] (B)	2/4 (50 %)	[97] (B) [94]	3/7 (43 %)
	−									[97] (G)	
	ND	[65]		[65]		[87]		[73] [90] (G)		[18, 58]	
Grade (school year level)	+							[99]		[98]	
	ND	[99]								[18]	
BMI			0		?		−		−−		−−
BMI/weight (higher)	−	[57, 71]	2/6 (33 %)	[57] [76] (G)	2/5 (40 %)	[71, 78] [64] (A5)	3/4 (75 %)	[19, 72, 73, 101]	4/4 (100 %)	[57, 61, 88, 94, 100] [76] (G)	6/10 (60 %)
	ND	[64, 75, 76, 78]		[64, 75] [76] (B)		[76]				[58, 75] [76] (B) [100] (Gr10B)	
Body fat/height/waist/weight											
Birth weight	ND					[81]	0/1				
Body fat	−									[84, 88]	2/2
Height	+		0/1		0/1	[64] (A3)	1/1				0/1
	ND	[64] (A4 and 5)		[64] (A4 and 5)						[88]	
Waist	−									[88, 100]	2/3
	ND									[100] (Gr8G, Gr10B)	
Advantage/disadvantage			?		+		+		?		+
Socioeconomic status	+	[66]	2/5 (40 %)	[66]	2/2 (100 %) (high SES) 1/1 (100 %) (low SES)	[71, 74]	3/4 (75 %)	[90] (G)	1/2 (50 %)	[66]	1/1 (100 %)
	−			[89]							
	ND	[71]						[90] (B)			
Region (two disadvantaged regions)	+			[22]							
	ND	[22]									
Social advantaged school	+	[83] (Gr1)				[83] (Gr1)					
	ND	[83] (Gr4)				[83] (Gr4)					
Sex			++		0		0 (B) ? (G)		++		?
Sex	+ (B)	[22, 23, 53, 56, 59, 62–65, 70, 75, 77, 88, 92, 96] [95] (A4–10)	16/26 (62 %) (B)	[23, 62]	2/20 (10 %) (B) 3/20 (15 %) (G)	[74] (high SES)	1/12 (8 %) (B) 5/12 (42 %) (G)	[19, 59, 80] [99] (preschool)	4/5 (80 %) (B)	[18, 62, 75, 88, 97, 98] [79] (score)	7/18 (39 %) (B) 2/18 (11 %) (G)
	+ (G)			[56, 70, 88]		[59, 67, 93] [74] (low SES) [83] (Gr4G)				[79] (time) [82] (G)	
	ND	[49, 50, 52, 57, 60, 66, 68, 99] [83] (Gr1 and 4) [95] (A11–12)		[22, 49, 50, 52, 53, 57, 60, 63–66, 68, 75, 89, 96]		[55, 64, 77, 92, 95] [83] (Gr1)		[99] (primary)		[52, 53, 56–58, 66, 69, 84, 94]	
Other											
Ethnicity	ND									[18, 82]	0/2
Fitness	+									[18]	1/1
Limb laterality	ND									[54]	0/1
**Behavioral attributes and skills**											
Activity class participation											
Dance classes	−	[49]	1/1		0/1						
	ND			[49]							
Kindy-gym classes	ND	[49]	0/1	[49]	0/1						
Swimming lessons	+		0/1	[49]	1/1						
	ND	[49]									
Physical activity			?		?		0		+		+
Active physical recreation	ND	[65]	5/11 (45 %)	[65]	5/11 (45 %)		0/1 (0 %)		¾ (75 %)		¾ (75 %)
Participation in physical activities	+	[65] (B)		[65] (B)							
	ND	[65] (G)		[65] (G)							
Physical activity	+	[33] (MVPA) [49] (MVPA)		[50] (cpm) [33] (MVPA) [51] (steps)						[84] [51] (steps) [97] (Gr9 all, Gr7B)	
	ND	[50] (cpm) [51] (steps)		[49]		[51] (steps)		[73]		[97] (Gr7)	
Organized sport participation	+	[65] (B)						[72, 90, 91]			
	ND	[65] (G)		[65, 89]							
Sports practice	+	[60] (G)		[60]							
Unstructured physical activity/leisure time	ND	[49]		[49]							
Sedentary time (less)	+							[80]	1/1		
Skills											
Early body control	+									[48]	1/1
Early hand control	−									[48]	1/1
Other											
Interactive game use	+	[50]	1/1		0/1						
	ND			[50]							
Non-interactive game use	ND	[50]	0/1	[50]	0/1						
**Cognitive, emotional and psychological factors**											
Cognitive											
Strategic knowledge	+									[18]	1/1
Infant factors											
APGAR at 5 min	ND					[81]	0/1				
Bailey infant behavior record	+					[81]	1/1				
Bailey mental scale	ND					[81]	0/1				
NBAS motoric cluster at 21 days	+					[81]	1/1				
Bailey motor scale	ND					[81]	0/1				
Psychological											
Perceived competence	+	[33]	1/1	[33]	1/1						
**Cultural and social factors**											
Adoption status	−					[87]	1/1				
Maternal education	+			[87]	1/1						
Mother with diabetes	−									[86]	1/1
Parental physical activity	ND	[49]	0/1	[49]	0/1						
Parent–child interaction	ND	[49]	0/1	[49]	0/1						
Parent skill confidence	+	[49]	1/1		0/1						
	ND			[49]							
Paternal education	ND			[89]	0/1						
Time living in USA	ND					[87]	0/1				
Time spent institutionalized before adoption	−					[87]	1/1				
**Physical environmental factors**											
Neurotoxicity											
Arsenic exposure	−					[85]	1/1				
Manganese exposure	ND					[85]	0/1				
Selenium exposure	ND					[85]	0/1				
Physical environment											
Playground at preschool (bigger)	+		1/1	[52]	1/1						0/1
	−	[52]									
	ND									[52]	
Toys and home equipment	ND	[49]	0/1	[49]	0/1						
Visits to play spaces	ND	[49]	0/1	[49]	0/1						

“0” = 0–33 %; “?” = 34–59 %; “+” = ≥60 % positive association; “−” = ≥60 % negative association; “++” = ≥60 % positive association with four or more studies; “−−” = ≥60 % negative association with four or more studies

*A* age, *APGAR* Appearance, Pulse, Grimace, Activity and Respiration, *B* boys, *BMI* body mass index, *cpm* counts per minute, *G* girls, *Gr* grade, *MVPA* moderate to vigorous physical activity, *NBAS* Brazelton Neonatal Behavioral Assessment Scale, *ND* non-determinant, *SES* socioeconomic status

### Meta-Analysis of Motor Competence Correlates

**Fig. 2:**
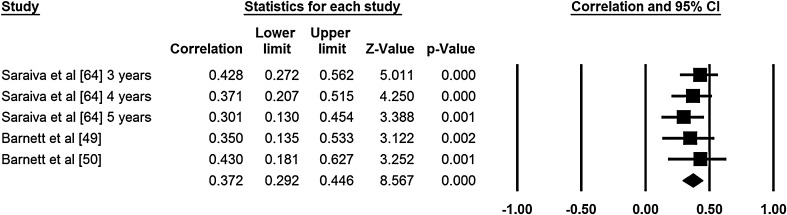
Meta-analysis of the relationship between age and object control movement skill competency; final row indicates overall correlation coefficient, which can be interpreted as an effect size estimate. *Q* = 1.584, *I* ^2^ = 0.000, *p* = 0.812, classic fail safe *N* = 90. *CI* confidence interval

**Fig. 3:**
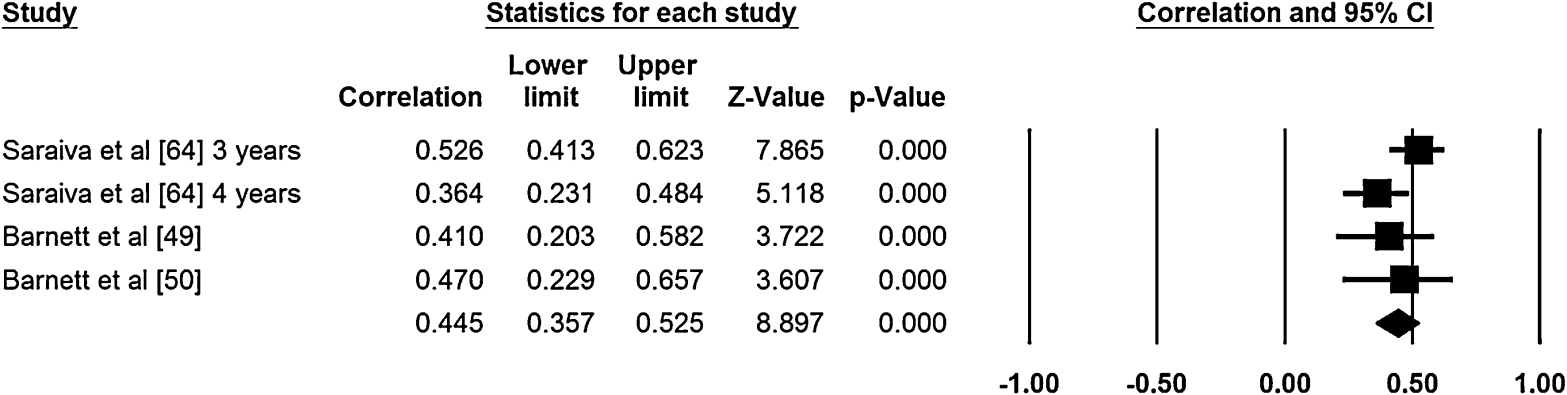
Meta-analysis of the relationship between age and locomotor movement skill competency; final row indicates overall correlation coefficient, which can be interpreted as an effect size estimate. *Q* = 3.913, *I* ^2^ = 23.382, *p* = 0.271, classic fail safe *N* = 104. *CI* confidence interval

**Fig. 4:**
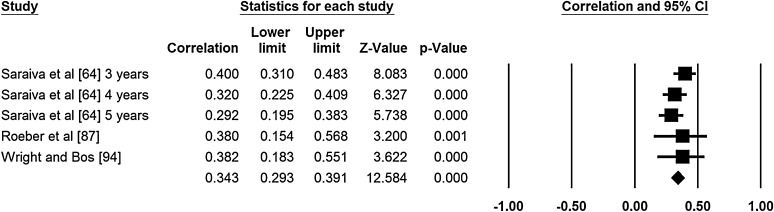
Meta-analysis of the relationship between age and stability; final row indicates overall correlation coefficient, which can be interpreted as an effect size estimate. *Q* = 3.287, *I* ^2^ = 0.000, *p* = 0.511, classic fail safe *N* = 185. *CI* confidence interval

**Fig. 5:**
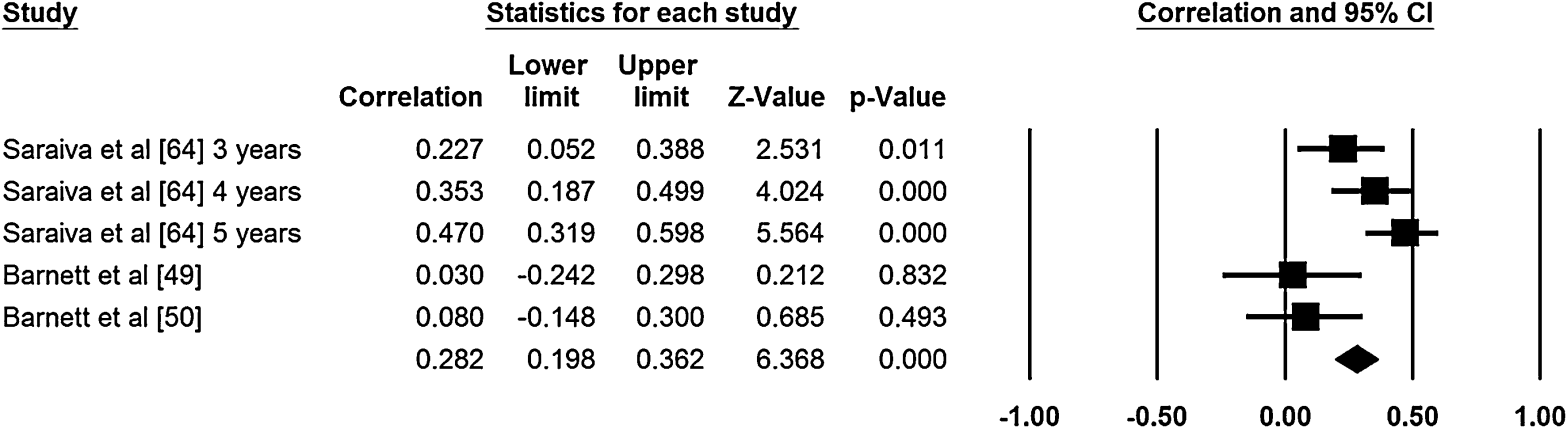
Meta-analysis of the relationship between sex and object control movement skill competency; final row indicates overall correlation coefficient, which can be interpreted as an effect size estimate. *Q* = 13.515, *I* ^2^ = 70.402, *p* = 0.009, classic fail safe *N* = 40. *CI* confidence interval

Two authors extracted data for the meta-analyses (NDR, ER). Meta-analyses were conducted for age and sex (Figs. 2, 3, 4, 5). No other correlates were investigated in three or more studies and reported standardized regression coefficients. The meta-analyses revealed small to medium effects for age and aspects of motor competence. For age, moderate effects were observed for object control skills [*r* = 0.37, 95 % confidence interval (CI) 0.29–0.35; Fig. 2, *Q* = 1.58, *I* ^2^ = 0.000, *p* = 0.812, classic fail safe *N* = 90], locomotor skills (*r* = 0.44, 95 % CI 0.37–0.51; Fig. 3, *Q* = 3.913, *I* ^2^ = 23.38, *p* = 0.271, classic fail safe *N* = 104), and stability skills (*r* = 0.34, 95 % CI 0.29–0.39; Fig. 4, *Q* = 3.29, *I* ^2^ = 0.000*, p* = 0.511, classic fail safe *N* = 185). A small effect was observed for the relationship between sex and object control skills, in favor of boys (*r* = 0.23, 95 % CI 0.09–0.36; Fig. 5, *Q* = 13.515, *I* ^2^ *=* 70.40, *p* = 0.009, classic fail safe *N* = 40). Low levels of heterogeneity were observed for the meta-analyses examining the relationship between skills and age. However, significant heterogeneity was found in the model that tested the relationship between sex and object control skills (*Q* = 13.515, *I* ^2^ = 70.402, *p* = 0.009). For all meta-analyses, a large number of studies with an effect size of zero (classic fail safe *N* values ranged from 40 to 185) would be required to cause the pooled point estimate to become statistically insignificant [46].

## Discussion

### Overview of Findings

It is clear from this review that investigating the correlates of gross motor competence is an emerging area, with the majority of studies (69 %) published in the last 5 years (since 2010). The most examined correlates of gross motor competency were biological and demographic factors, with age (positive), sex (boys more skilled than girls for object control and motor coordination), and adiposity (negative for motor coordination, stability, and skill composite) identified as correlates. In the behavioral attributes and skills category, PA and sport participation were the most investigated correlates, with some evidence for PA being a positive correlate of motor competence. Only one study examined cognitive, emotional and psychological factors as correlates of motor competence [18], precluding any conclusions regarding these outcomes. Similarly, only four studies [49, 86, 87, 89] investigated cultural and social factors that might contribute to motor competence, with mixed results. Finally, only three studies [49, 52, 85] investigated physical environment factors.

This review included only those studies for which gross motor competence was chosen as the outcome variable for the analysis, and therefore we did not include studies in which motor competence was a predictor or in which a simple bivariate analysis was conducted. With 83 % (49/59) of the studies being cross-sectional, it could be argued that this is a matter of semantics, as either variable could be placed as the outcome. However, this assumption is not strictly correct. To illustrate, Barnett and colleagues [33], in a cross-sectional study, examined reciprocal associations between motor competence and PA in adolescents, using path analysis, and found a reciprocal relationship between object control and moderate to vigorous physical activity (MVPA) and a one-way relationship from MVPA to locomotor skills. Restricting our included studies in this way is a study strength as we have isolated studies that hypothesized motor competence as the outcome in our effort to better understand what factors potentially influence motor competence. This does not mean that cross-sectional evidence can be regarded as causal, but rather that the variables have been analyzed according to our hypothesis of interest.

Our meta-analyses of biological factors revealed small-to-medium effects for age and motor competence. The only other systematic review in this area (in preschool children only) also identified biological/demographic variables (such as sex and age) as having an association with motor competence [31]. Our meta-analysis showed that age was positively associated with locomotor, object control, and stability skills. It is not surprising that the older a child is, the better their skills, provided they continue to have opportunities to participate in activities that build competence. Motor development in young children in the very early years is more influenced by biological maturation, and after this period, it becomes influenced more by practice and opportunity. Thus it is feasible that the relationship between age and gross motor competence might change across the developmental periods of early childhood, preschool, childhood and adolescence. Interestingly, even though the summary evidence confirmed age as a positive correlate of most aspects of motor competence, there were some studies (across all types of motor competence) that did not find this to be the case. For instance, when skill composite was the outcome in seven studies that investigated age, three reported a positive association, one study found a negative association for girls and three studies found no association. The study that found age to be a negative correlate was in the adolescent age group. This study suggested that girls’ decline in their motor competence was due to reduced opportunity to be active, as the study also found that girls’ PA declined during this period [97].

The null age findings in the other studies could be because many instruments (e.g., the TGMD) [102] provide the ability to age-standardize scores, so if these scores are being used in analyses, there may not be an age effect after standardizing. An alternate explanation for age not consistently being a correlate may be that some instruments used to assess motor competence have a ceiling effect and therefore fail to identify age differences in the older age groups. For example, instruments designed to assess motor competence via process-oriented assessments in young children have fewer and simpler assessment components than those designed for older children. Hence there may potentially be a certain age threshold where a motor competence assessment tool is no longer appropriate and a more complex assessment instrument is required. This underscores the need to ensure the instrument has been validated among the age group it purports to assess. This would suggest that the studies in older children and adolescents would be less likely to show positive associations between age and gross motor competence. Yet of the studies that found age was not a correlate for at least one aspect of motor competence, two were in the early age groups (preschool), where skill trajectories are greater [58, 65], five were in primary/elementary school children [18, 73, 87], and none were conducted in adolescents. It would therefore be unlikely that the children in these studies have all reached their maximum skill level or that the instruments used to assess motor competence had a ceiling effect. Furthermore, a different instrument to assess motor competence was used in each of these five studies, precluding the ability to find a pattern due to instrumentation. It is more likely that the age range investigated was not wide enough to show differences by age; this appears to be the case for four of the five studies [18, 58, 65, 87].

Weight status had differential associations with aspects of gross motor competence. Higher BMI was negatively correlated with motor coordination, stability, and skill composite, but not with object control skill competency. An indeterminate association was found for locomotor skills. An inverse relationship between body weight status and motor competence (defined broadly) has been found in other reviews, but these reviews did not examine associations for weight status with different categories of gross motor competence [2, 11]. In contrast to object control skills, which tend to be more static, locomotor and stability skills involve shifting or controlling a larger body mass, which impedes functional movement [103] and contributes to the higher rate of lower limb problems among obese children (e.g., tibia varus, plantar pressure) [104]. The negative association between composite gross motor competence scores and higher BMI could reflect the composition of assessments where the composite comprises more motor coordination while moving and controlling the body compared with object control skills assessments.

Similarly, the sex of a child as a correlate of gross motor competence was also equivocal; sometimes males were favored, sometimes there were indeterminate results, and many studies showed no associations. This uncertainty is probably due to the fact that sex appears to relate differently to various aspects of gross motor competence. Being male was found to be a strong positive correlate of object control competence and motor coordination tasks. The meta-analyses confirmed this for object control skills, although the effect size was small. It is possible there is a biological basis for boys being more competent in object control skills. Butterfield et al. [105] allude to evolutionary/biological differences pre-maturation for boys and girls, especially in reference to skills such as throwing and striking. Size and power might also be reflected in these findings, although considering only seven of the 59 studies included adolescents (and thus included males likely to have matured), this is unlikely to be the reason. The Iivonen and Sääkslahti [31] review also found being male to be a positive correlate for object control skills in the preschool age group, providing further support that size and strength due to maturation may not explain these findings. Product-oriented assessments may be favorable towards size and power as they are concerned with the outcome of the movement (i.e., how far, how high), rather than the process of the movement. Although, of the ten studies that did not find sex to be a correlate of object control competence, seven used a process-oriented assessment [49, 50, 52, 57, 60, 66, 68] and three used a product-oriented assessment [83, 95, 99], so there is no clear pattern favoring one type of assessment instrument over another. It is also likely that sociological factors may explain the difference in object control competence between girls and boys. Numerous studies have demonstrated that, compared with girls, boys receive greater encouragement, support and opportunities in PA and sports at home and in school and the broader community. As a result, girls’ opportunities to enhance their gross motor competence may be limited, which would result in widening the gender gap [106–111]. Different PA and sport preferences between girls and boys may also help to explain this sex difference in object control skills. Being male is also a consistent positive correlate of PA in young children (aged 4–9 years) and a correlate for older children and adolescents [112].

We also found that sex was not a correlate of locomotor skills and had an indeterminate association for females for stability. In contrast, the Iivonen and Sääkslahti [31] review found that being female was a positive correlate for balance and locomotor skills. The three studies in our review that did find being female was a positive correlate for locomotor skills were all focused on children, but not as young as preschool children (ranged from 5–8 years) [56, 70, 88]; however, other studies with children of a similar age did not find a sex effect for these types of skills. A potential explanation for different findings between the reviews is that whilst the Iivonen and Sääkslahti [31] review found four studies that indicated locomotor skills were better performed by girls, this review only included studies that did find an association. Therefore these three studies may not represent the findings of the breadth of studies in the field.

We found that socioeconomic advantage was a positive correlate of locomotor skill stability and skill composite. Disadvantaged children may have less home sports equipment, reduced parental support and finances for organized sport and therefore be limited in terms of developing particular skills [113, 114]. There is a positive association between family support and PA in children and adolescents. Similarly, in adolescents, general social support for PA has been identified as a correlate [112].

Different aspects of PA and sport participation constituted the majority of investigations in the behavioral attributes and skills category. Interestingly, whilst we found PA to be a positive correlate of skill composite and motor coordination, we also found indeterminate evidence for PA being a correlate of object control or locomotor skill competence. Iivonen and Sääkslahti [31] found habitual PA to be a correlate of motor competence in preschool children, as have other reviews [2, 10] with PA (as the outcome). However these previous reviews did not attempt to see if different constructs of motor competence were predictive of PA. Our results suggest the relationship between PA and gross motor competence is not straightforward. It has been suggested that the relationship between motor competence and PA is not completely reciprocal [10]. In the model by Stodden et al. [12], it is postulated that in young children, PA is important to build motor competence but as children age, motor competence becomes more important for PA participation. Consequently, the relationship between PA and motor competence might vary depending on age of the child. It is logical that different types of PA may have differing associations with skills. It is also feasible that participation in the types of activities that use particular skills may lead to higher associations with that type of skill competence. For example, high participation in track events in athletics would likely be associated with better locomotor skills. Whilst we grouped PA factors together so as to provide a summary score, it is possible that if types of PA have differing associations with skill competence, these differences will be masked. One study was included that investigated different types of class participation in young children (dance, “kindy gym,” swimming) and found the association was different according to object control and locomotor competence, which supports this hypothesis [49]. However, because only one study did this, summary scores could not be calculated. When examining PA intensity, rather than type of activity participation, there is also evidence (when PA intensity is the outcome) of differing associations. For example, MVPA and vigorous physical activity are commonly associated with motor competence [2, 33], whereas the few studies that investigate light activity did not find a relationship [115–117]. We would hypothesize a negative relationship between sedentary time and motor competence, and this was confirmed in the one study that investigated this [80]. Future research may seek to further investigate the nuances of the relationship between PA and gross motor competence to be able to tease out exactly what sorts of activity better contribute to what sorts of motor competence (and the reverse) at different ages. In order to understand the association between PA and motor competence, more appropriate and informative measures are required, particularly in children. Objective methods such as accelerometry, pedometers and global positioning systems are not yet sophisticated enough to comprehensively document the quality, context and type of activity. For example, some of the active play movements may register very little on an accelerometer (e.g., climbing, crawling, etc.). Direct observation would provide this information, but it is only a snapshot and costly.

There is a wide scope for future researchers to replicate studies and attempt to find evidence, particularly in the areas of cognitive, emotional and psychological factors. The link between cognition, PA and fitness has gained some attention recently, and this has extended to motor competence [118–120]. We only had one study that examined these aspects as correlates of motor competence [18], indicating this as an area of future research need. One study investigated infant factors (e.g., infant behavior record was a positive correlate) as predictors of motor competence in a longitudinal study [81]. This sort of investigation is quite unique in the literature but crucial to identifying the early life factors that contribute to better motor competence.

Only four studies investigated cultural and social factors that might contribute to motor competence, with these factors based on the parent [e.g., parent confidence (positive), mother with diabetes (negative)] or the child [e.g., adoption status (negative)]. One large study of correlates of gross motor competence in preschool children could not be included in this review as they used an assessment tool that includes fine motor skills in its composite score and thus did not meet our eligibility criteria [121]. Cools et al. [121] investigated the effects of a number of family and neighborhood characteristics on gross motor competence and identified factors such as father’s PA and transport to school by bicycle as having a positive influence. This study [121] also identified some family factors negatively associated with preschool children’s gross motor competence. Even though there is currently limited evidence in this area to draw any conclusions, the existing studies do point to the worthiness of future research in this area.

Only three studies in this review investigated physical environment factors (including such diverse factors as neurotoxicity, physical space, toys and equipment). Some positive and negative correlates were identified, again reinforcing the need for further research in this area.

### Strengths and Limitations

This systematic review is the first to our knowledge that has investigated correlates of gross motor competence in children and adolescents. Using gross motor competence as a global definition is a study strength as a large number of studies could be included. A further strength is that we categorized correlates according to the specific ways motor competence has been defined and operationalized (object control, motor coordination, etc.), which enables us to have a greater understanding of which correlates influence specific types of motor competence. Indeed our findings do suggest that, once summary scores of motor competence are considered, evidence for some correlates differs according to how motor competence is operationalized. This shows that if we are seeking to provide a ‘full’ assessment of a child’s motor competence, instruments should be used that can cover the broad spectrum of motor competence from motor coordination to fundamental movement skills. A recent validity investigation confirms this approach, finding that the KTK and the TGMD (version 2) measure discrete aspects of motor competence [122].

As stated in Sect. 4.1, it is a study strength that we isolated studies that hypothesized motor competence as the outcome in our effort to better understand what factors potentially influence motor competence. It is a further strength of this review that we conducted meta-analyses. However, very few studies focused on the same correlate and the same motor skill outcome, which meant we were limited by a lack of data for inclusion. Also, very few studies provided regression coefficients, and because of our exclusion criteria regarding correlation analysis, correlation data could not be used in our meta-analyses. Nevertheless we were able to provide support for some of the summary findings with regard to age and sex, which strengthens our results.

## Conclusions

Age (increasing) is a correlate of children’s gross motor competence. Weight status (healthy), sex (male) and socioeconomic background (higher) are consistent correlates for certain aspects of motor competence only. “Being male” as a correlate of object control skills and motor coordination has important intervention implications, as there is growing evidence of object control competence being a more salient predictor of PA and fitness behavior than locomotor competence [14, 70, 123]. Boys consistently have higher object control competence, which is a concern for females as their PA declines more than boys over adolescence, so if their object control competence is also lower, they may experience a negative spiral of engagement, ultimately resulting in an unhealthy weight status [12].

Somewhat in contrast to other reviews, we did not find PA to be a consistent positive predictor of motor competence [2, 10]. The hypothesized Stodden model suggests that children who engage in more PA develop better motor competence and fitness and that this positive spiral of engagement ultimately impacts on weight status [12]. A narrative review has since examined the current state of evidence to support the hypothesized Stodden model [8]. Their conclusion was that the latest evidence indicates that motor competence is positively associated with multiple aspects of health (i.e., PA, cardiorespiratory fitness, muscular strength, muscular endurance and a healthy weight status). Based on the evidence in this current review, we can confirm that both PA and weight status are important to motor competence, but this relationship does appear to depend on the way motor competence is operationalized. This finding also has important intervention implications, suggesting that addressing childhood and adolescent obesity prevention through motor competence interventions, such as those tested in after-school settings [124], requires further investigation.

The authors of the aforementioned narrative review also concluded that there are still questions remaining related to the increased strength of associations across time and in terms of the direction of associations (i.e., what is the antecedent and what is the consequent [8]). This current systematic review has contributed to this understanding by specifically highlighting the factors that predict gross motor competence. Future researchers may seek to investigate the role of many correlates of motor competence that could not be evaluated due to the small number of studies for each correlate, so as to build the knowledge base in this area.
